# For Eczema of the Scalp

**Published:** 1886-10

**Authors:** 


FOR ECZEMA OF THE SCALP.—

| | | | |
|---|---|---|---|
| ℞ | Acidi salicylici, - - - - | gr. viii. | |
| | Spts. Mindereri, - - - - | gr. xxv. | |
| | Glycerini, - - - - | ℥xii. | M. |

FOR SCABIES.

| | | | |
|---|---|---|---|
| ℞ | Napthol, - - - - | | |
| | Chalk, - - - - - - | | |
| | Sulphur Precip. aa, - - - - | gr. x | |
| | Lard, - - - - - | ℥ xii. | M. |

—*Journal Cutan. and Vener. Diseases.*

MARCHI, of Florence, has studied the degenerations following total or partial extirpation of the cerebellum. Total extirpation produces a diffuse sclerosis of the gray substance surrounding the pyramidal bodies, atrophy of the transverse fibres of the middle peduncle of the pons varolii, sclerosis of the gray substance of the olivary bodies, and degeneration of the cerebellar peduncles. Extirpation of half the cerebellum produces a sclerosis of the fibres of the opposite half of the pons and medulla oblongata; extirpation of the middle lobe of the cerebellum produces a bilateral degeneration, confined to certain fibres.

---

MEDICAL JOURNALISM IN TEXAS.—"Our worthy President, Dr. E. P. Baxter, emphatically *saw* the theological *ante* of Brother Brown and '*went him considerably better.*' Indeed, Dr. Brown *went it blind*, as to the sentiments of the members, and Dr. Baxter certainly *straddled the blind* when he said: — — — — This declaration of the president of the Texas Medical Association amounts to an emphatic *call*, and hence this article, a *showing of hands*, as it were."—*Daniels' Texas Medical Journal.* While Dr. Daniels' theology and medical philosophy are excellent, we fear he did not acquire his peculiar literary style in the pulpit.

